# Neurocomplications of Recreational Drug Use

**DOI:** 10.5334/jbr-btr.1436

**Published:** 2017-11-18

**Authors:** Luc van den Hauwe, Jef Huyskens, Simon Nicolay, Johan W. Van Goethem, Paul M. Parizel

**Affiliations:** 1 Antwerp University Hospital, BE

**Keywords:** brain, hemorrhage – emergency imaging

Drug abuse is a substantial problem in society and is associated with significant morbidity and mortality. It’s an epidemic that crosses racial, socioeconomic, and age barriers. According to the 2016 Global Drug Survey the top 10 drugs – excluding alcohol, tobacco and caffeine products – used across the world are: cannabis, MDMA, cocaine, amphetamines, LSD, magic mushrooms, prescribed and non-prescribed opioid medication, nitrous oxide, ketamine, and poppers.

Various drugs may cause central nervous system (CNS) complications. These complications include acute and chronic neurovascular complications, toxic leukoencephalopathy, atrophy, infection, changes in the corpus callosum, and other miscellaneous changes.

The lecture will focus on neurovascular complications which constitute the most frequent drug related emergencies. They include both ischemic stroke, subarachnoid, and intracerebral hemorrhage.

Also nonischemic white matter changes, i.e. toxic leukoencephalopathy, has been described with some drugs. A more nonspecific leukoencephalopathy is seen in chronic inhalant abuse of industrial solvents such as toluene. Such lesions can be explained by the high affinity of these lipophilic volatile agents for myelin.

